# Toscana Virus Infection Imported from Elba into Switzerland

**DOI:** 10.3201/eid1606.091763

**Published:** 2010-06

**Authors:** Martin Gabriel, Christiane Resch, Stephan Günther, Jonas Schmidt-Chanasit

**Affiliations:** Bernhard Nocht Institute for Tropical Medicine, Hamburg, Germany (M. Gabriel, S. Günther, J. Schmidt-Chanasit); Basel University Medical Clinic, Liestal, Switzerland (C. Resch)

**Keywords:** Viruses, Toscana virus, vector-borne infections, Elba, Switzerland, meningitis, sandfly, arboviruses, PCR, letter

**To the Editor:** Toscana virus (TOSV) is a serotype of *Sandfly fever Naples virus* (SFNV) within the family *Bunyaviridae* and the genus *Phlebovirus*. TOSV is transmitted to humans by sandflies (*Phlebotomus* spp.) and is a prominent cause of aseptic meningitis in Mediterranean countries (*1*). In Italy, for populations living in rural areas and persons engaging in outdoor activities, the highest risk for acquiring TOSV is from August through October (*1*). TOSV infections should therefore be considered in travelers returning from the Mediterranean area who have fever and signs of meningitis. Laboratory diagnosis of TOSV infections is often limited to the detection of immunoglobulin (Ig) M and IgG because of the short period of viremia and the low amount of virus in the cerebrospinal fluid (CSF) during the acute phase (*2*). We report a reverse transcription–PCR (RT-PCR)–confirmed TOSV infection acquired on the island of Elba that was then imported into Switzerland.

A 17-year-old man was referred to Basel University Medical Clinic, Liestal, Switzerland, in August 2009 with headache, recurrent episodes of vomiting, photophobia and phonophobia, and an elevated temperature of 38.1°C. The patient had returned to Switzerland from a vacation on the island of Elba, Italy, 14 days before. He recalled that he had received multiple insect bites on the beach. Cardiopulmonary and neurologic examination showed tachycardia and nuchal rigidity. Results of a complete blood count and liver and kidney function tests showed no abnormalities. CSF analysis showed lymphocytic pleocytosis (47 cells/µL), and aseptic meningitis of viral origin was suspected. Empirical treatment with acyclovir (2.3 g/day) was started for the first 48 hours. The results of a PCR for herpesviruses were negative in the CSF sample, and serologic testing showed no evidence of acute infection with herpesviruses. CSF, urine, and blood cultures showed negative results for fungi and bacteria, including mycobacteria. The patient did not show signs of immune deficiency.

Serum and CSF samples were sent to the Bernhard-Nocht-Institute for Tropical Medicine in Germany for SFNV diagnostics. Results of immunofluorescent assays for TOSV and SFNV were positive with IgM titers of 1,280 and 160, respectively (cut-off 20) and IgG titers of 5,120 and 640, respectively (cut-off 20). Real-time RT-PCRs for detection of TOSV and SFNV were performed using the CSF sample according to a recently published protocol (*3*). A positive result was obtained for TOSV, and the PCR result was confirmed by sequencing the PCR product. Phylogenetic analysis demonstrated that the TOSV from Elba clustered with the TOSV A lineage (Figure). Attempts to isolate TOSV from the CSF sample in cell culture failed. The patient was afebrile on the second day of hospitalization, headache vanished on the third day, and he was discharged on day 5 restitutio ad integrum (fully recovered).

**Figure:**
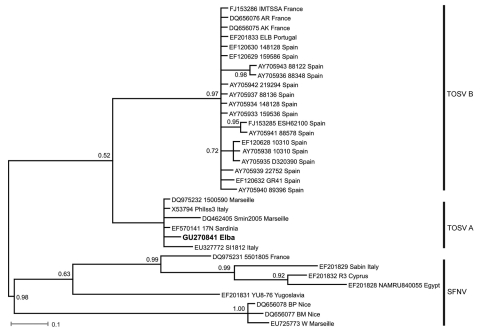
Bayesian phylogenetic tree of Toscana virus (TOSV) and *Sandfly fever Naples virus* (SFNV) strains. For each sequence used, GenBank accession number, strain designation, and strain origin are shown. Phylognetic analysis was performed by using MrBayes 3.0 program (*4*) with a general time reversible substitution model. Substitution rates were assumed to follow a gamma plus invariants distribution. Three heated chains and a single cold chain were used in all Markov Chain Monte Carlo analyses, which were run for 1,000,000 generations, sampling 1 tree every 100 generations. Trees obtained before convergent and stable likelihood values were discarded (i.e., a 2,500 tree burn-in). Four independent runs, each started from different, randomly chosen trees, were performed to assess convergence. Posterior probabilities for nodes were assembled from all post burn-in trees (i.e., 30,004 trees per analysis). Posterior probabilities are shown on each node. Scale bar indicates nucleotide substitutions per site. The newly described TOSV sequence from Elba is shown in **boldface**.

This report demonstrates the presence of TOSV on the island of Elba by molecular detection and typing. This finding is in agreement with previous serologic reports on imported TOSV infections from this area into central Europe (*5**,**6*). However, because of serologic cross-reactivity, serologic tests are usually not able to clearly discriminate between TOSV and other SFNV infections (*2*).

Real-time RT-PCR is the most appropriate tool for the differentiation of TOSV from other SFNV infections and enables molecular typing of amplified sequences. The Bayesian phlyogenetic tree calculated with the short PCR fragment (111 bp, GenBank accession no. GU270841) of the nucleocapsid coding sequence (Figure) shows the same topology of the main clades when compared with trees obtained with the complete N coding sequence (*7**,**8*). The assignment of the TOSV from Elba to lineage A (Figure) is consistent with results of previous studies, demonstrating that this is the dominant genotype of TOSV in mainland Italy and the island of Sardinia (*8*).

The presence of TOSV A on the island of Elba is a major public health issue for the local population and for the >2 million tourists that visit Elba every year (*9*). Given the incidence of TOSV infections in other surrounding Mediterranean countries, one could assume that the virus is present in other islands of the Mediterranean, posing a public health problem for the resident population and tourists alike. Molecular and serologic surveillance studies in Mediterranean countries could identify potential high risk areas for TOSV infections to help prevent exposure of local residents and tourists to the virus. Moreover, the risk of transfusion-associated transmission of arboviruses in European countries should be addressed.
